# The Role of Scavenger Receptor B1 in Infection with *Mycobacterium tuberculosis* in a Murine Model

**DOI:** 10.1371/journal.pone.0008448

**Published:** 2009-12-24

**Authors:** Georgia Schäfer, Reto Guler, Graeme Murray, Frank Brombacher, Gordon D. Brown

**Affiliations:** 1 Division of Immunology, Institute for Infectious Diseases and Molecular Medicine (IIDMM), University of Cape Town, Cape Town, South Africa; 2 International Centre for Genetic Engineering and Biotechnology (ICGEB), Cape Town, South Africa; 3 Section of Translational Medicine, Division of Applied Medicine, School of Medicine and Dentistry, University of Aberdeen, Foresterhill, United Kingdom; 4 Section of Immunology and Infection, Division of Applied Medicine, Institute of Medical Sciences, University of Aberdeen, Foresterhill, United Kingdom; Institut Pasteur, France

## Abstract

**Background:**

The interaction between *Mycobacterium tuberculosis* (Mtb) and host cells is complex and far from being understood. The role of the different receptor(s) implicated in the recognition of Mtb in particular remains poorly defined, and those that have been found to have activity *in vitro* were subsequently shown to be redundant *in vivo*.

**Methods and Findings:**

To identify novel receptors involved in the recognition of Mtb, we screened a macrophage cDNA library and identified scavenger receptor B class 1 (SR-B1) as a receptor for mycobacteria. SR-B1 has been well-described as a lipoprotein receptor which mediates both the selective uptake of cholesteryl esters and the efflux of cholesterol, and has also recently been implicated in the recognition of other pathogens. We show here that mycobacteria can bind directly to SR-B1 on transfected cells, and that this interaction could be inhibited in the presence of a specific antibody to SR-B1, serum or LDL. We define a variety of macrophage populations, including alveolar macrophages, that express this receptor, however, no differences in the recognition and response to mycobacteria were observed in macrophages isolated from SR-B1^−/−^ or wild type mice *in vitro*. Moreover, when wild type and SR-B1^−/−^ animals were infected with a low dose of Mtb (100 CFU/mouse) there were no alterations in survival, bacterial burdens, granuloma formation or cytokine production in the lung. However, significant reduction in the production of TNF, IFNγ, and IL10 were observed in SR-B1^−/−^ mice following infection with a high dose of Mtb (1000 CFU/mouse), which marginally affected the size of inflammatory foci but did not influence bacterial burdens. Deficiency of SR-B1 also had no effect on resistance to disease under conditions of varying dietary cholesterol. We did observe, however, that the presence of high levels of cholesterol in the diet significantly enhanced the bacterial burdens in the lung, but this was independent of SR-B1.

**Conclusion:**

SR-B1 is involved in mycobacterial recognition, but this receptor plays only a minor role in anti-mycobacterial immunity *in vivo*. Like many other receptors for these pathogens, the loss of SR-B1 can be functionally compensated for under normal conditions.

## Introduction

Tuberculosis is the leading cause of death worldwide from a single infectious disease. The causative agent, *Mycobacterium tuberculosis* (Mtb), enters the host typically via aerosols, and alveolar macrophages are considered the first cells to engulf Mtb and become infected. Although the initial interaction of the pathogen with the host macrophage is considered a critical step in the pathogenesis of Mtb, the role of the receptors that play a role in mediating the entry of Mtb into macrophages and in transducing intracellular signals is very controversial and far from being understood. Over the years, more than a dozen receptors have been shown to recognize and bind mycobacteria (reviewed in [1]). However, the role of these receptors has been mostly based on *in vitro* examination in transfected cells; studies using inhibitors or animals deficient for specific receptors have indicated that these receptors are dispensable [2]–[7]. Since no direct functional-based search for the macrophage receptors involved in mycobacterial recognition has been performed, here we utilised a generalized screening method [8] with which we identified scavenger receptor B class 1 (SR-B1) as a novel macrophage receptor involved in the recognition of Mtb.

Scavenger receptors belong to a family of cell surface transmembrane glycoproteins with broad ligand-binding abilities and important roles in atherogenesis, innate immunity and macrophage regulation (reviewed in [9]). Although their impact for recognition of Mtb has not been extensively studied, there are a few reports that show the general involvement of scavenger receptors in binding of mycobacteria [2], [10]. Recently, the scavenger receptors MARCO and SR-A were shown to be involved in binding of cord factor [11].

Scavenger receptor B1, in particular, has been well-described as a lipoprotein receptor which is expressed primarily in liver and nonplacental steroidogenic tissues, and mediates both the selective uptake of cholesteryl esters and the efflux of cholesterol [12]–[14]. A member of the CD36 family of scavenger receptors, SR-B1 consists of 509 amino acids and is expressed as a 82kDa glycoprotein with two cytoplasmic C- and N-terminal domains separated by a large extracellular domain [15]. This receptor has been shown to interact with both native and chemically modified (oxidized and acetylated) low-density lipoprotein (LDL), high-density lipoprotein (HDL), very-low-density lipoprotein and anionic phospholipids [12], [16]–[18]. Accumulating evidence suggests that the function of SR-B1 as well as other members of the CD36 family is not solely linked to cholesterol metabolism but involves a wide spectrum of activities, including microbial recognition. For example, the human homologue CLA-1 as well as the *Drosophila* homologue Pes have been shown to mediate binding and uptake of various bacteria, including both Gram^+^ and Gram^−^ organisms [19], [20] with Pes being the only member of the CD36 family so far that has been described to be involved in mycobacterial recognition [19]. Furthermore, SR-B1 was shown to mediate the entry of hepatitis C virus (HCV) in an HDL-dependent manner [21], [22], while it also plays an important role in the infection of hepatocytes by the malaria parasite [23].

Here we have identified SR-B1 as a receptor for mycobacteria, and show that it can mediate the binding of mycobacteria *in vitro*. However, we found that SRBI plays only a minor role *in vivo* and that its function can be compensated for by other macrophage receptors.

## Results

### Identification of SR-B1 as a Macrophage Receptor Involved in Binding of Mycobacteria

**Figure 1:**
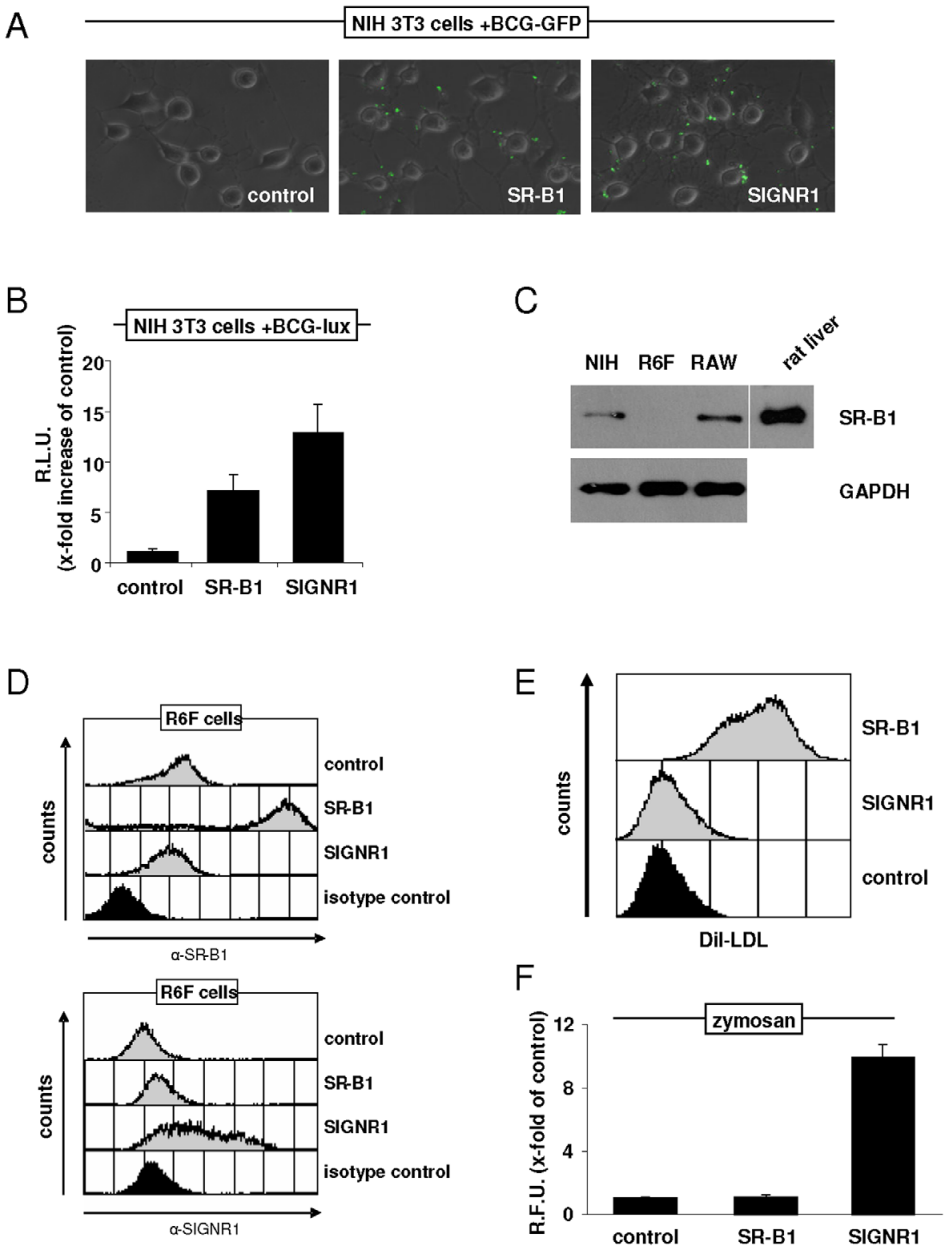
Identification of SR-B1 as a receptor for mycobacteria. NIH3T3 cells stably transfected with empty vector pFBneo (negative control), SR-B1 or SIGNR1 (positive control), respectively, were incubated with BCG-GFP (A) or BCG-lux (B) and assessed for binding by fluorescence microscopy or luciferase activity, respectively. Shown is the x-fold increase of luciferase activity compared to vector control which was set as 1. Experiments were performed in triplicate and normalised to cell number by CFSE staining. (C) Western Blot showing expression of SR-B1 in various untransfected cell lines as indicated. Cellular lysates from rat liver were included as a control, and GAPDH served as loading control. (D) FACS analysis of R6F cells stably transduced with pFB (vector control), SR-B1 or SIGNR1, and stained with anti-SR-B1 or anti-SIGNRI, as indicated. (E) FACS assay showing binding/uptake of DiI-LDL to R6F cells stably expressing pFB (vector control), SR-B1 or SIGNR1. (F) Binding of FITC-labelled zymosan to R6F cells stably expressing pFB (vector control), SR-B1 or SIGNR1, as quantified by fluorometry. Shown is the x-fold increase of fluorescence compared to vector control which was set as 1. Experiments were performed in triplicate and normalised to cell number by CFSE staining.

To identify new receptors involved in binding and recognition of mycobacteria, a retroviral cDNA expression library, generated from RAW264.7 macrophages [24], was stably expressed in NIH3T3 cells and visually screened by fluorescent microscopy for binding of live *Mycobacterium bovis* bacillus Calmette-Guérin expressing Green Fluorescent Protein (BCG-GFP). Positive cells were isolated and enriched in culture until almost pure colonies of BCG-binding cells were obtained. After isolation of their genomic DNA and re-amplification of the stably inserted cDNA fragments originating from the RAW264.7 library, a 2.5kb cDNA fragment corresponding to the full-length murine scavenger receptor B class 1 (SR-B1) was obtained that was tested positive for BCG-GFP binding when re-inserted into NIH3T3 cells (Fig. 1A). When quantified for mycobacterial binding using luciferase expressing BCG cells (BCG-lux) the SR-B1 expressing NIH3T3 revealed a 7-fold increase in BCG binding compared to vector control cells (Fig. 1B). To include a positive control for BCG-binding, NIH3T3 cells stably expressing SIGNR1 [25] were used, and these cells showed slightly more mycobacterial binding of about 12-fold versus controls (Fig. 1A and B).

### Characterization of Mycobacterial Binding to SR-B1 In Vitro Using Transfected Cells

To test BCG binding in cells which do not express any endogenous SR-B1, we screened several cell lines by Western blotting. Using this approach, we found that R6F cells were negative for SR-B1, while both NIH3T3 cells and RAW264.7 macrophages expressed this receptor (Fig. 1C). We therefore generated R6F cells stably expressing SR-B1 or SIGNR1 (as a positive control) and tested their receptor expression by FACS (Fig. 1D). These cells were also functionally tested for binding of known ligands for the respective receptors, including DiI-labeled LDL (Fig. 1E, [16]), for SR-B1, and zymosan (Fig. 1F, [25]), for SIGNRI.

**Figure 2:**
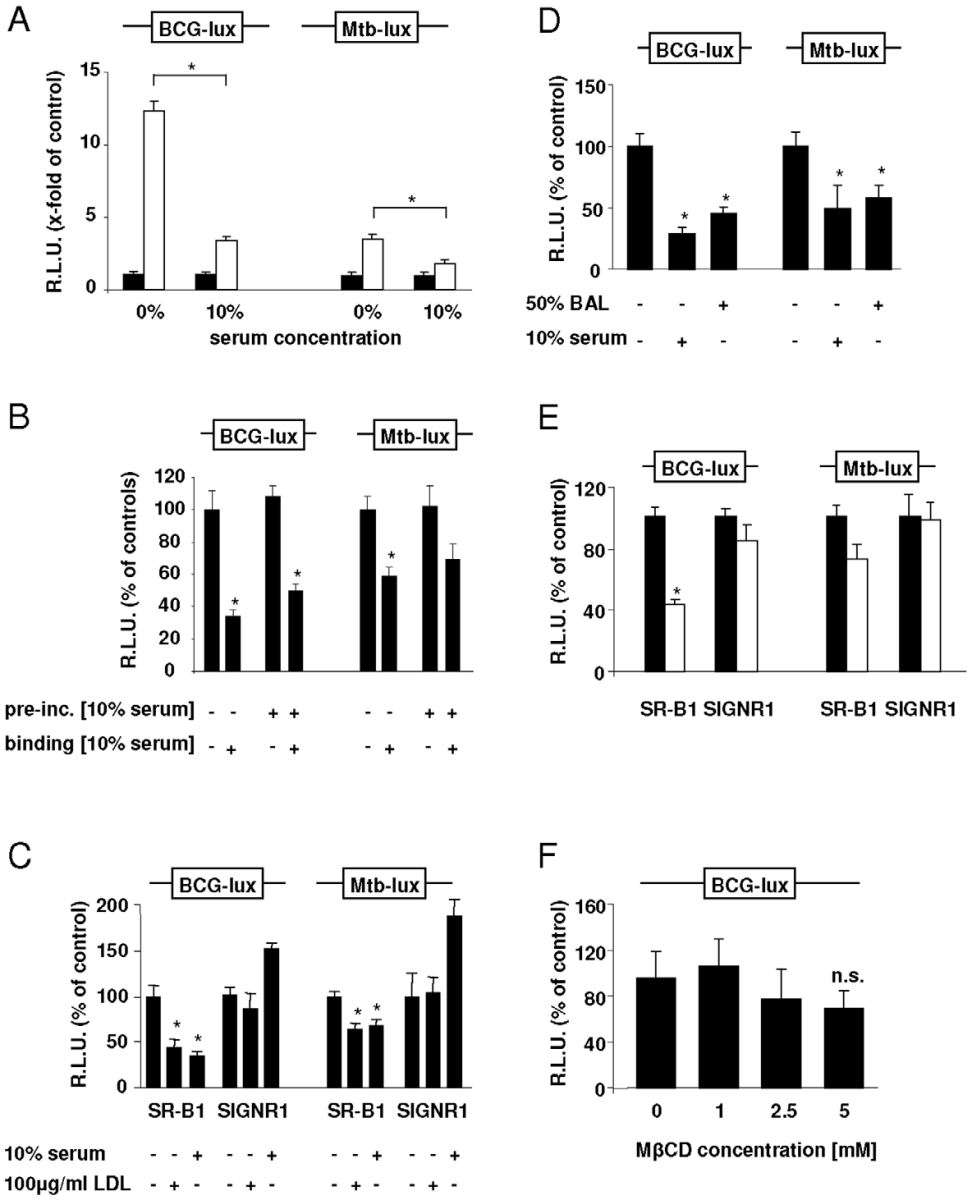
Characterization of BCG and Mtb binding to SR-B1 transfected cells. R6F cells stably transfected with the indicated constructs were incubated with either BCG-lux (left panels) or Mtb-lux (right panels), and quantified for binding by measuring luciferase activity. Experiments were performed in triplicate and normalised to cell number by CFSE staining. (A) Quantification of mycobacterial binding to SR-B1 expressing cells (white bars) or vector control cells (black bars) in the presence or absence of serum, as indicated. Shown is the x-fold increase of luciferase activity compared to vector control which was set as 1. (B) Mycobacteria (BCG-lux or Mtb-lux) were pre-incubated in medium with or without 10% serum, prior binding to R6F-SR-B1 cells also in the presence or absence of 10% serum. Shown is % of luciferase activity relative to binding to R6F-SR-B1 cells in the absence of serum, which was set at 100%. (C) Effect of LDL on binding of mycobacteria to R6F cells expressing SR-B1 or SIGNR1, as indicated. Shown is % of luciferase activity relative to R6F-SR-B1 or R6F-SIGNR1 cells, respectively, in the absence of additives. (D) Effect of BAL fluid and serum (as a control) on binding of BCG-lux or Mtb-lux to SR-B1 expressing R6F cells. Shown is % of luciferase activity relative to R6F-SR-B1 in the absence of additives. (E) Effect of anti-SR-B1 antibodies on binding of BCG-lux or Mtb-lux to SR-B1 or SIGNR1 transfected R6F cells. The white bars show % of luciferase activity relative to binding in the absence of antibody (black bars). (F) SR-B1 expressing R6F cells were incubated with mycobacteria in the presence of increasing concentrations of MβCD. Binding of BCG-lux is shown as % of luciferase activity relative to control (no MβCD).

SR-B1 is a lipoprotein receptor, and we observed a significant decrease in the binding of BCG-lux to R6F cells expressing SR-B1 when binding was performed in the presence of serum (Fig. 2A, left panel). Notably, binding of mycobacteria to SR-B1 transfected cells, although reduced, still occurred in the presence of serum. To further investigate whether the serum-inhibition of bacterial binding was direct or indirect, we performed experiments where BCG-lux was pre-incubated in medium in the presence or absence of serum and then added to SR-B1 expressing cells in the presence or absence of serum (Fig. 2B, left panel). The bacteria pre-incubated in the absence of serum were inhibited by serum, as observed before (see Fig. 2A, left panel). A similar effect was observed when the bacteria were pre-incubated in the presence of serum; binding of these bacteria was only inhibited by the presence of serum during the binding assay. Importantly, pre-incubation of the bacteria in serum did not affect subsequent binding to the SR-B1 expressing cells in the absence of serum. Therefore the serum-inhibition effect was due to direct competition of serum components for the receptor rather than an “opsonization” effect on the bacteria. Based on this finding, all later experiments were performed in the absence of serum.

To further demonstrate this competition, we performed binding experiments in the presence of LDL in serum-free medium. Although SR-B1 is known to have broad ligand specificity [15], and recognizes various different lipoprotein classes, we chose unmodified LDL as this particle has shown to bind specifically to SR-B1 but not CD36 or class A scavenger receptors [16]. As shown in Fig. 2C (left panel), both LDL and serum significantly blocked binding of BCG-lux to SR-B1 expressing R6F cells, while this effect was not observed in R6F cells expressing SIGNR1. We also observed that bronchoalveolar lavage containing lung surfactant significantly blocked binding of BCG-lux to SR-B1 transfected cells (Fig. 2D).

As mentioned above, SR-B1 is well characterised regarding its role in cholesterol metabolism. Also, it is known that mycobacteria can bind to free cholesterol in the membrane [26]. Therefore experiments were designed to investigate whether the observed binding of BCG-lux to cells expressing SR-B1 was due to direct interaction of the bacteria with the receptor, or because of an increased accumulation of cholesterol in the membrane. To this end, the binding of BCG-lux to SR-B1 was investigated after blocking of the receptor with a specific antibody. As shown in Fig. 2E (left panel) SR-B1 expressing R6F cells pre-incubated with anti-SR-B1 showed a significant reduction in binding of BCG-lux, whereas the antibody had no affect on the binding of the mycobacteria to control SIGNR1 cells. This was further supported by the observation that depletion of the membrane's cholesterol by cyclodextrins did not significantly reduce the amount of BCG-lux binding to R6F cells expressing SR-B1 (Fig. 2F), although SR-B1 transfected R6F cells did show more cholesterol accumulation when stained with filipin compared to control cells (data not shown).

We next wanted to test whether these results also held true for virulent *Mycobacterium tuberculosis* H37Rv. Although we had some difficulty in generating uniform suspensions of this bacterium which interfered with our binding assays, all the trends observed with BCG-lux could be confirmed with Mtb (Fig. 2A–E; right panels). Importantly, Mtb-lux bound to SR-B1 transfected R6F cells and this binding could be inhibited by the presence of serum, LDL and BAL (Fig. 2A, C and D, right panels), which was due to direct competition for the receptor (Fig. 2B, right panel). Further, the binding of Mtb-lux to SR-B1 expressing cells could be inhibited in the presence of an antibody against SR-B1 (Fig. 2E, right panel). Thus overall, these data suggest that SR-B1 can directly recognize mycobacteria and that this interaction can be inhibited by serum and surfactant components.

### The Role of SR-B1 for Mycobacterial Infection of Primary Cells

**Figure 3:**
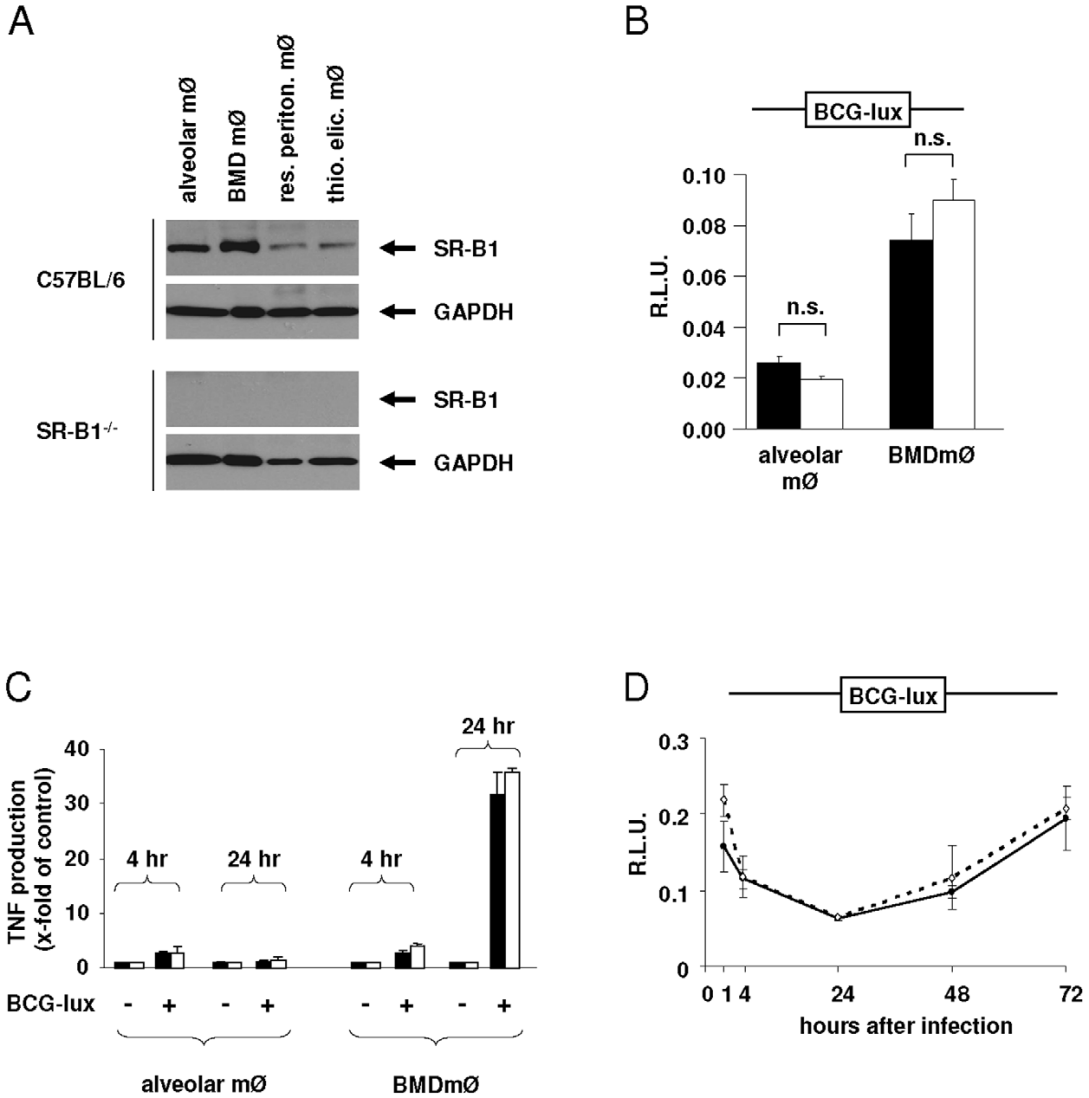
The role of SR-B1 in mycobacterial recognition in primary cells. (A) Western Blot assessing expression of SR-B1 in different macrophage populations derived from wild type C57/BL6 and SR-B1^−/−^ mice, as indicated. (B) Alveolar macrophages and BMDmØ isolated from wild type (black bars) and SR-B1^−/−^ mice (white bars) were tested for BCG-lux binding in the absence of serum. Shown is the relative luciferase activity (R.L.U.), normalised to cell number by CFSE staining. (C) TNF production at 4 hr or 24 hr after binding of BCG-lux to alveolar macrophages or BMDmØ isolated from wild type (black bars) and SR-B1^−/−^ mice (white bars). Shown is x-fold increase in TNF production relative to control (no BCG binding). Experiments were normalised to cell number by CFSE staining. (D) Survival of BCG-lux in infected BMDmØ isolated from wild type (solid lines) and SR-B1^−/−^ mice (dashed lines). After infection of macrophages, unbound mycobacteria were removed and samples taken after the indicated time points to assess luciferase activity. Experiments were performed in triplicate, normalised to cell number by CFSE staining and shown as R.L.U. relative to time point 1.

Having demonstrated that SR-B1 can mediate the recognition of mycobacteria in transfected cells, we next investigated the contribution of this receptor on primary cells. Initially, different macrophage populations (alveolar macrophages, bone-marrow derived macrophages (BMDmØ), resident as well as thioglycollate-elicited peritoneal macrophages) were isolated from wild type C57BL/6 mice and tested for the expression of SR-B1 by Western Blotting (Fig. 3A). This analysis demonstrated the expression of this receptor on all macrophage populations examined, and was absent in macrophages derived from SR-B1^−/−^ mice. Importantly, alveolar macrophages and BMDmØ expressed highly SR-B1, therefore these cell populations were tested for binding of BCG-lux (Fig. 3B). Surprisinlgy, no statistically significant differences in binding could be observed between wild type and SR-B1^−/−^ macrophages in the absence of serum. Notably, mycobacterial binding to resident alveolar macrophages was inefficient when compared to BMDmØ, as has been demonstrated in a previous study [27].

When analysed for TNF production 4 hr or 24 hr after mycobacterial binding, no differences between wild type and SR-B1^−/−^ macrophages were detected (Fig. 3C). Although the level of TNF was equally low after 4 hr in both macrophage populations, it increased considerably in BMDmØ after 24 hr, presumably reflecting the differences in bacterial binding as described above. These experiments were repeated with varying parameters in bacterial binding, including different incubation times and temperatures, but no differences between wild type and SR-B1^−/−^ macrophages were detected under any of the conditions tested (data not shown). We also found that the presence of serum inhibited binding of mycobacteria to BMDmØ equally well in both wild type and SR-B1^−/−^ macrophages, while the addition of MβCD (to deplete cholesterol membrane) had no effect on binding (data not shown). Furthermore, when we blocked SR-B1 with a specific antibody, binding of mycobacteria to the macrophages was unaltered compared to untreated controls (data not shown). Finally we investigated whether the survival of mycobacteria in macrophages was affected by the presence or absence of SR-B1. To this end, wild type and SR-B1^−/−^ BMDmØ were infected with BCG-lux and mycobacterial viability analysed over time by measuring luciferase activity. As shown in Fig. 3D, both wild type and SR−B1^−/−^ macrophages could equally efficiently kill mycobacteria within the first 24 hr after infection, following which the bacteria started to replicate. Thus loss of SR-B1 has minimal impact on mycobacterial recognition by primary macrophages *in vitro*.

### SR-B1 Is Dispensable for Infection with *Mycobacterium tuberculosis* In Vivo

**Figure 4:**
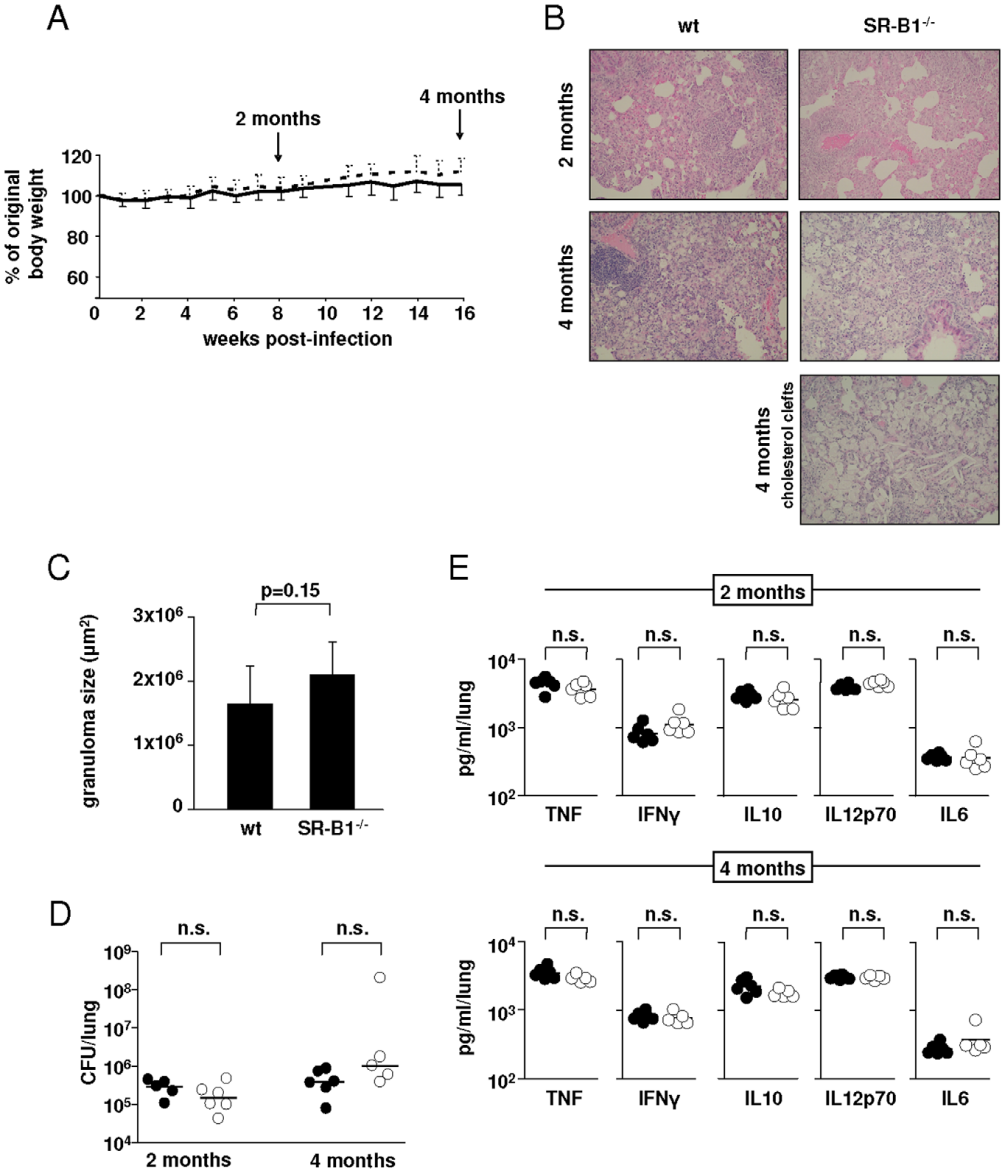
The role of SR-B1 *in vivo* following low dose infection with Mtb. Wild type C57/BL6 and SR-B1^−/−^ mice were infected with 100 CFU *Mycobacterium tuberculosis* H37Rv by aerosol route and sacrificed after 2 and 4 months. (A) Body weight was monitored throughout the course of the experiment and is presented as % of original body weight (time of infection) with the SEM shown as solid lines for wild type animals and dashed lines for SR-B1^−/−^ mice. Lungs of infected animals were analysed at 2 and 4 months for histopathology by H&E staining (B). Also shown is a lung section showing the presence of clefts of accumulated cholesterol that were observed in some inflammatory foci in infected SR-B1^−/−^ mice. Lung sections after 4 months of infection were further anlaysed by morphometric analysis to calculate the sizes of the inflammatory lesions (C). Lung homogenates were analysed at 2 and 4 months for bacterial burden (D), as well as the production of TNF, IFNγ, IL10, IL12p70 and IL6 (E) with the black circles representing wild type and the open circles representing SR-B1^−/−^ animals. Shown are the data from individual mice and the median value.

To explore a potential role for SR-B1 *in vivo*, wild type and SR-B1^−/−^ mice were aerosol infected with 100 CFU *Mycobacterium tuberculosis* H37Rv, and the development of disease monitored after 2 and 4 months. As shown in Fig. 4A, both wild type and SR-B1^−/−^ mice survived and gained weight throughout the course of the infection. The lungs of both groups displayed inflammatory foci, consisting of patchy infiltrates of inflammatory cells, predominantly foamy macrophages (Fig. 4B). Typical granulomas were not observed. Some SR-B1^−/−^ mice showed evidence of clefts of accumulated cholesterol, which were not observed in the wild type animals (Fig. 4B and data not shown). When the sizes of inflammatory foci were measured by morphometric analysis (Fig. 4C), SR-B1^−/−^ mice did not show significant differences to the wild type mice. Furthermore, no differences in bacterial burdens (Fig. 4D) or production of TNF, IFNγ, IL10, IL12p70 or IL6 (Fig. 4E) were detected between wild type and SR-B1^−/−^ mice.

**Figure 5:**
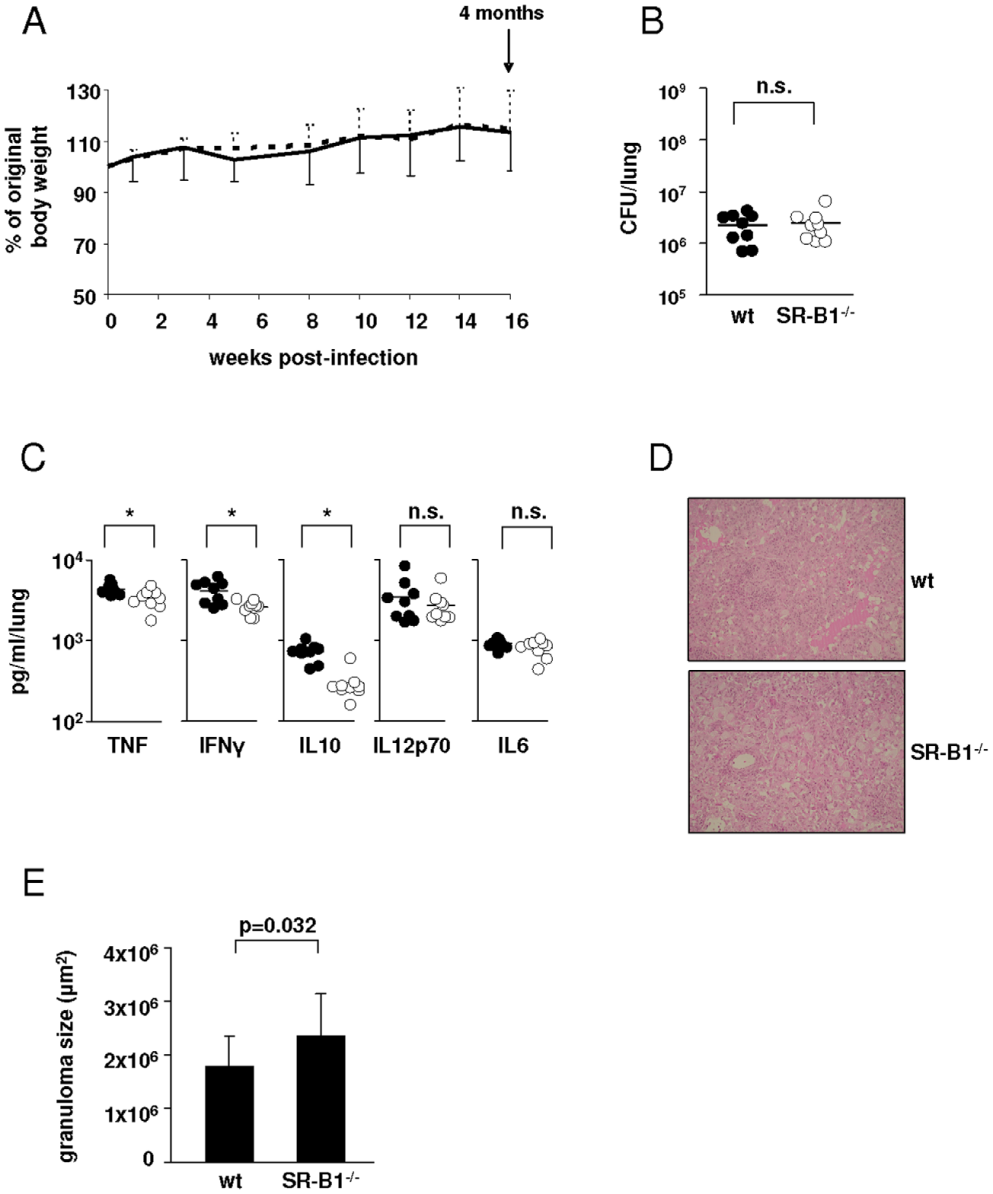
The role of SR-B1 *in vivo* following high dose infection with Mtb. Wild type C57/BL6 and SR-B1^−/−^ mice were infected with 1000 CFU *Mycobacterium tuberculosis* H37Rv by aerosol route and sacrificed after 4 months. (A) Body weight was monitored throughout the course of the experiment and is presented as % of original body weight (time of infection) with the SEM shown as solid lines for wild type animals and dashed lines for SR-B1^−/−^ mice. Lungs of infected animals were analysed for bacterial burden (B), TNF, IFNγ, IL10, IL12p70 and IL6 (C), histopathology (D) and inflammatory lesion size (E), with the black circles representing wild type and the open circles representing SR-B1^−/−^ animals. Shown are the data from individual mice and the median value. *, p<0.05.

We also tested the effects of infection with a high dose of Mtb, i.e 1000 CFU per lung (Fig. 5). As before, both groups of mice gained weight and did not display any adverse effects during the course of the infection (Fig. 5A). Although we detected significantly less pulmonary TNF, IL10 and IFNγ in the SR-B1^−/−^ animals (Fig. 5C), this did not correlate with differences in bacterial burdens (Fig. 5B). Although the inflammatory pathology was similar between both groups of mice, except for the presence of occasional cholesterol clefts (Fig. 5D and data not shown), the size of the inflammatory foci in the SR-B1^−/−^ mice were found to be slightly increased (p-value of 0.032) (Fig. 5E).

**Figure 6:**
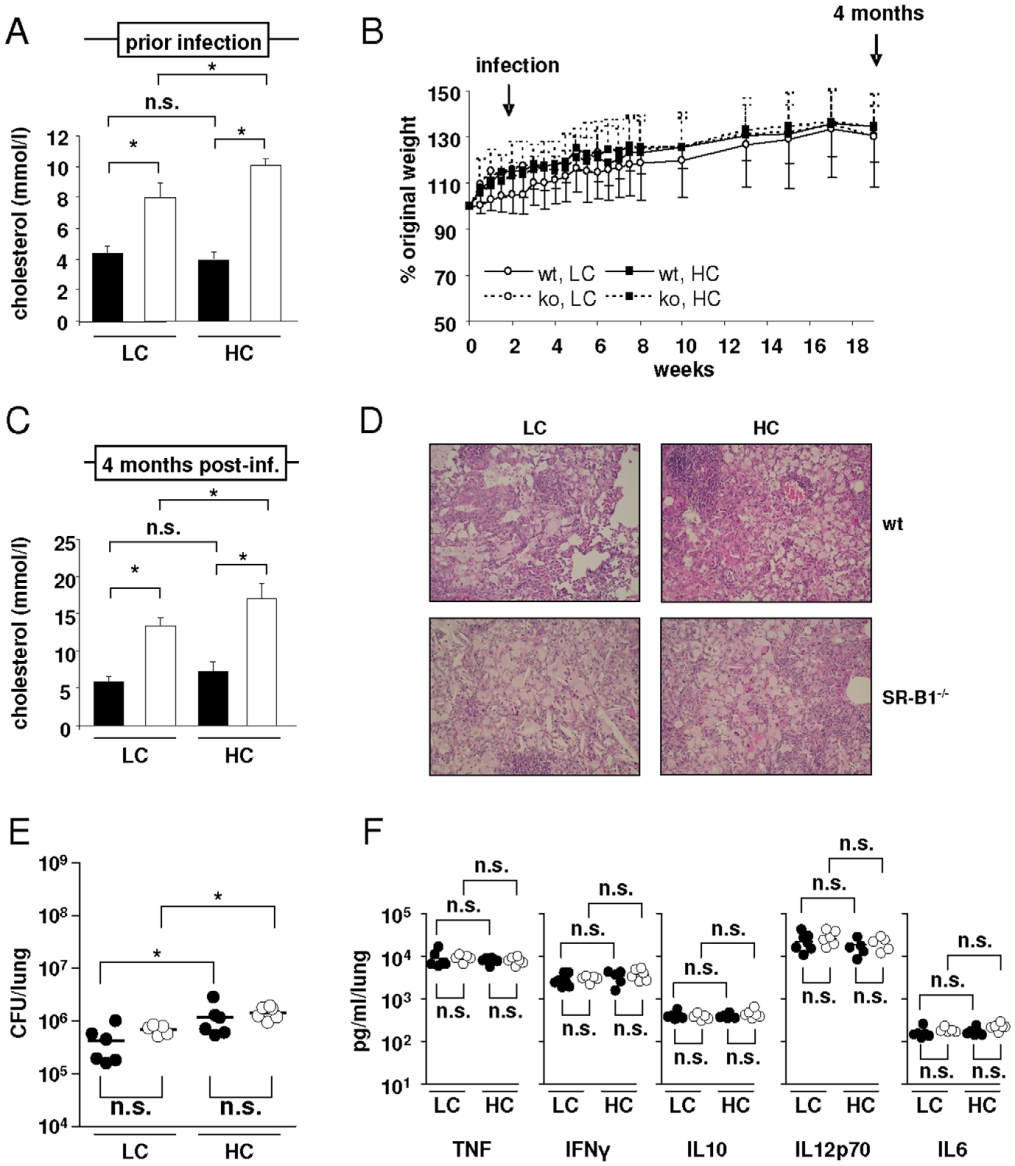
The effect of cholesterol in SR-B1^−/−^ mice during Mtb infection. Wild type C57/BL6 and SR-B1^−/−^ mice were fed either a low cholesterol diet (LC, 0.15% cholesterol) or a high cholesterol diet (HC, 1.25% cholesterol) throughout the experiment, infected with 100 CFU *Mycobacterium tuberculosis* H37Rv by aerosol route and sacrificed after 4 months. (A) Levels of serum cholesterol following 2 weeks on the various diets, as indicated, prior to infection (black bars representing wild type, and white bars the SR-B1^−/−^ animals). (B) Average mouse weight throughout the course of the experiment, presented as % of original body weight ±SEM. 4 months after infection the mice were analyzed for serum cholesterol (C), histopathology (D), bacterial burdens (E), and pulmonary TNF, IFNγ, IL10, IL12p70 and IL6 (F) with the black circles representing wild type and the open circles representing SR-B1^−/−^ animals. Shown are the data from individual mice and the median value. *, p<0.05.

Elevated serum cholesterol levels are known to correlate with susceptibility to tuberculosis [28], and as SR-B1 is involved in cholesterol metabolism, we assessed the influence of cholesterol in the diet on susceptibility to mycobacterial infection in wild type and SR-B1^−/−^ mice. Appropriate numbers of animals were fed either a low cholesterol (LC, 0.15% cholesterol) or a high cholesterol (HC, 1.25% cholesterol) diet and then infected with 100 CFU Mtb. Cholesterol levels at the time of infection were significantly higher in SR-B1^−/−^ mice compared to wild type mice on either diet, and higher levels of cholesterol were detected in SR-B1^−/−^ mice on the HC diet compared to animals on the LC diet (Fig. 6A). As observed in our previous experiments (Fig. 4A and 5A), all animals gained weight and did not show any adverse effects during the course of the infection (Fig. 6B). When the experiment was terminated after 4 months, there were similar relative differences between the various mouse strains in serum cholesterol levels, although the SR-B1^−/−^ mice showed an overall general increase (Fig. 6C, compare with Fig. 6A). We also observed a slight, but significant, diet-dependent difference in bacterial burden of the lungs, with elevated bacterial levels in both wild type and SR-B1^−/−^ mice on the HC diet compared to the corresponding animals on the LC diet (Fig. 6E). However, there was no difference in bacterial burden between wild type and SR-B1^−/−^ mice on either diet (Fig. 6E). Despite the slightly elevated bacterial burdens in mice on HC diets, there were no significant differences in histopathology, except for the occasional presence of cholesterol clefts in SR-B1^−/−^ lungs (Fig. 6D), or in the levels of pulmonary cytokine (Fig. 6F).

## Discussion

The identification of host pattern recognition receptors that recognise Mtb represents a potential opportunity for anti-mycobacterial prophylaxis, as the interruption of binding may functionally disrupt Mtb entry into its host cell niche. By screening a retroviral cDNA expression library derived from RAW264.7 murine macrophages, we identified SR-B1 as a receptor for mycobacteria (Fig. 1A and B). As a member of the CD36 family of scavenger receptors, which have been well described for their function in cholesterol metabolism, we show here that SR-B1 is also involved in mycobacterial recognition. To our knowledge, this activity so far has only been attributed to the *Drosophila* homologue Pes [19].

As SR-B1 is involved in cholesterol transport across the membrane [12]–[14] and as mycobacteria have been shown to display high binding capacity for free cholesterol [26], we wondered if the expression of SR-B1 would lead to an accumulation of cholesterol in the membrane and indirectly lead to increased mycobacterial binding. However, depletion of the membrane's cholesterol by cyclodextrins did not significantly reduce mycobacterial binding to SR-B1 expressing cells (Fig. 2F). This is supported by an earlier study on SR-B1 expression of transfected COS-7 cells, where the presence of SR-B1 had no effect on the level of free cholesterol [29]. We also demonstrated that mycobacteria bound directly to SR-B1, and that this binding could be competitively inhibited by specific antibodies, lipids, serum and surfactant (Fig. 2A–E).

While we clearly show that SR-B1 is involved in recognition of mycobacteria on transfected cells we could not define a role for this receptor in primary cells, although SR-B1 was clearly expressed in wild type macrophages (Fig. 3A) as has been shown earlier for the macrophage cell line RAW264.7 [30]. While disappointing, this was not entirely unexpected, as another study investigating the human homologue of SR-B1, was shown to recognise Gram positive and Gram negative bacteria in transfected, but not in primary cells [20].

Studies on primary macrophages revealed no difference in binding of mycobacteria and subsequent TNF production in the presence or absence of SR-B1 (Fig. 3B and C). However, binding of mycobacteria to both wild type and SR-B1^−/−^ BMDmØ was much stronger than to alveolar macrophages (Fig. 3B), and this is likely to be due to the expression of other recognition receptors on these cells, as has been shown previously [11], [31]. We also found that serum blocked binding of mycobacteria to both wild type and SR-B1^−/−^ alveolar macrophages and BMDmØ (data not shown) as opposed to an earlier observation where serum components increased binding to BMDmØ [31]. Furthermore, depletion of the membrane's cholesterol with MβCD had no effect on binding (data not shown), which is in contrast to a previous study [26]. This can be due to different macrophage populations, preparation techniques and different sera used. Also, other macrophage receptors might be more important for mycobacterial binding which render the role of the cholesterol content in the membrane redundant.

Our *in vivo* studies on the role of SR-B1 during mycobacterial infection also revealed no substantial difference between wild type and SR-B1^−/−^ mice when infected with either a low or a high dose Mtb or when investigated in the context of dietary cholesterol (Fig. 4D, Fig. 5B, Fig. 6E). However, we did observe some clefts of accumulated cholesterol, limited largely to the peripheral parenchyma, in the absence of SR-B1 after Mtb infection (Fig. 4B). The accumulation of cholesterol in the inflammatory lesions of the SR-B1^−/−^ mouse lungs might account for the fact that cholesterol transport across the macrophage membrane is impaired in these animals. Although we did detect decreased levels of TNF, IFNγ and IL10 in the SR-B1^−/−^ animals infected with a high dose Mtb (Fig. 5C), the bacterial burdens in both groups of animals was comparable (Fig. 5B). In fact, there were still substantial amounts of TNF and IFNγ present in the SR-B1^−/−^ lungs: 76% TNF and 63% IFNγ levels compared to those found in wild type animals, while the amount of IL10 was decreased to 40% compared to wild type. While the changes in cytokine levels are likely to have contributed to the slight difference in size of the inflammatory lesions that were observed following infection with a high dose Mtb (Fig. 5E), we do not feel these data represent a significant biological difference between wild type and knockout animals.

The lack of a substantial effect of SR-B1 deficiency was surprising, however, considering the elevated serum cholesterol levels in SR-B1^−/−^ mice (Fig. 6A and C). Indeed, a recent report demonstrated that high serum cholesterol levels in apolipoprotein E (ApoE) deficient mice fed on a HC diet, resulted in high susceptibility of to Mtb [28], although ApoE appeared to have a more pronounced influence on the regulation of plasma cholesterol than SR-B1. However, there were significantly increased bacterial numbers in the lungs of infected wild type and SR-B1^−/−^ animals fed on the HC diet compared to mice on the LC diet (Fig. 6E), which was independent of SR-B1, and, interestingly, did not appear to correlate with the serum cholesterol levels (Fig. 6C and 6E). Although not detectable in the serum, animals fed the HC diet might still have elevated intracellular cholesterol stores which can be taken up and used by Mtb as a source of energy to their growth advantage [32]. This might explain why those animals on the HC diet displayed higher bacterial burdens in the lungs compared to those fed a LC diet. However, the role of SR-B1 for mycobacterial growth seems to be minor and independent of the diet.

While playing a role for recognition of mycobacteria in an isolated context using transfected cells, our data supports the notion that macrophage receptors show redundancy when investigated in primary cells [1], [33]. This is true, for example, with the mannose receptor (MR), which has long been considered to play a central role in mycobacterial phagocytosis [34], and for the TLRs, which have recently emerged to contribute to the innate recognition of these pathogens [35], [36]. The situation is even more complex *in vivo*. Receptors such as CR3, for example, which is able to mediate both complement-opsonized as well as non-opsonized phagocytosis of mycobacteria *in vitro* on transfected cells [37] was not found to play a role *in vivo* [4]. Similarly contradicting findings have been described for the TLRs: although important *in vitro* [38], TLR2, TLR4, or mice triply deficient in TLR2, TLR4 and TLR9 [5], [6], [39] were infected with mycobacteria, no susceptibility to Mtb was observed. However, these data were contradicted by other groups showing that TLR2 and TLR4 do play roles in controlling the infection with Mtb [40], [41]. This clearly shows that when studying the interaction of Mtb with its host *in vivo* the particular experimental setting greatly affects the outcome.

In this context, the preparation of the mycobacteria has much influence on the experimental observations: we and others found that mycobacteria bind to and activate macrophages to a much greater extent when sonicated rather than syringed ([42] and data not shown). Also, when performing the binding assay under shear conditions rather than static binding one would simulate the dynamic and physiological conditions in the lung much better and select for higher-affinity receptors [43]. With the static binding conditions as presented here, one would also identify low-affinity receptors. It is possible that SR-B1, being a true scavenger with broad ligand specificity, is a low-affinity receptor for mycobacteria and simply plays only a minor role *in vivo*. Experimental differences will also occur when different strains of mycobacteria (virulent vs. avirulent) and/or genetic background of the receptor-deficient mice (relatively resistant such as C57BL/6 vs. relatively susceptible such as BALB/c) are being used. To add to the complexity, different macrophage populations (i.e. resident vs. elicited vs. immune-activated as well as human vs. murine) express a different array of receptors [11], [27] and therefore respond differently to infection with Mtb. This is also important in our experimental approach: we identified SR-B1 when screening a cDNA library generated from the murine macrophage cell line RAW264.7. These cells might express a completely different array of receptors compared to primary cells, leading to the identification of Mtb-binding surface molecules that might not be relevant *in vivo*.

As discussed in the literature before, it seems that Mtb enters the host cell via a number of possible receptor/ligand interactions [1], [33]. The ability of Mtb to engage multiple receptors (or receptor clusters) simultaneously might be an evolutionary advantage for its pathogenesis: in the absence of a single receptor, phagocyte infection by mycobacteria can be efficiently supported by alternative receptors.

Taken together, we demonstrate that SR-B1 is another example of the diverse spectrum of receptors that recognize mycobacteria *in vitro* but is redundant in the extremely complex macrophage-mycobacterium interaction *in vivo*. On the one hand, this redundancy of SR-B1 in mycobacterial infection might be a result, at least in part, of surfactant blocking the bacterial-receptor interactions, as we demonstrated *in vitro* (Fig. 2D). Surfactant may directly compete with Mtb for the receptor binding site, or that aggregated mycobacteria alter their interaction with the host cells, as shown for SP-D [44], [45]. On the other hand, the redundancy may stem from down-regulation of this receptor during mycobacterial infection, as macrophage activation has been shown to suppress SR-B1 expression [30]. Furthermore, expression of the related human scavenger receptor CD36 has been found to be lower on monocytes in TB patients, and reverse to normal levels upon anti-mycobacterial treatment [46]. At the same time, when resident alveolar macrophages differentiate by cytokine stimulation, they increase the expression of surface receptors and show a higher affinity to bind mycobacteria [27], therefore maybe completely render any role of SR-B1 redundant.

## Materials and Methods

### Cell Culture

NIH3T3 murine embryonic fibroblasts (ATCC), RAW264.7 murine macrophages (ATCC), rat 6 fibroblasts (R6F, [47]) and the packaging cell line plat-E [48] were grown in DMEM (Invitrogen) supplemented with 10% heat-inactivated fetal calf serum (Delta Bioproducts), 2mM L-glutamine, 100U/ml penicillin and 100µg/ml streptomycin (Sigma) in a humidified atmosphere (5% CO_2_) at 37°C, if not otherwise indicated.

### Ligand Binding Assays

The cells used for the binding assays were plated in 24-well-plates at a density of 5×10^4^ cells per well and allowed to adhere overnight. Nonopsonic binding studies were performed with live strains of *Mycobacterium bovis* bacilli Calmette-Guérin (BCG) expressing either Green Fluorescent Protein (BCG-GFP) or bacterial luciferase (BCG-lux) as well as *Mycobacterium tuberculosis* H37Rv expressing bacterial luciferase [49] (Mtb-lux) and FITC-labeled zymosan (Molecular Probes). Mycobacteria were grown in Middlebrook 7H9 (Difco) with 10% OADC (oleic acid, albumin, dextrose, catalase) enrichment in the presence of 10µg/ml kanamycin (BCG-GFP) or 50µg/ml hygromycin (BCG-lux), respectively, and kept as 15% glycerol stocks at −80°C at a density of approximately 5×10^6^ particles. For binding studies, BCG or Mtb aliquots were thawed, spun in a microcentrifuge for 1 min at 13000*g*, washed twice in DMEM followed by vigorous vortexing, and aggregates were dispersed by sonication for 30 sec at 50W in a cuphorn sonicator. To infect cells, culture medium was removed, and mycobacterial suspension in serum-free DMEM (if not otherwise indicated) was added at a particle∶cell ratio of 10∶1. Where indicated, binding was performed in the presence of additives such as 10% serum, 1∶100 diluted rabbit anti-mouse SR-B1 polyclonal antibody (NB 400-134, Novus Biologicals), 100µg/ml LDL, 50% bronchoalveolar lavage fluid (BAL), or methyl-β-cyclodextrin (MβCD, Sigma), respectively, at the indicated concentrations. FITC-labeled zymosan was prepared similarly, sonicated for 3 min at 150W and added to the indicated cells at a particle∶cell ratio of 10∶1. The cells were then incubated with their ligands for 1 hr at 37°C, washed three times with DMEM and prepared for further analysis.

### Quantification of BCG- and Zymosan Binding

Binding of BCG-lux to the cells was determined by lysis of the cells in 150µl 0.1% triton X-100 and addition of 20µl 1% n-decyl-aldehyde to initiate the luciferase reaction. Light emission was measured in white 96-well plates for 2.5 sec using a Fluoroscan Ascent FL (Thermo Fisher Scientific, Waltham, MA). Binding of FITC-zymosan was quantified by lysis of the cells in 100µl 3% triton X-100. The relative fluorescence was then determined in black 96-well plates using the Fluoroscan Ascent FL. Both BCG- and zymosan binding data were normalized to cell numbers by fluorescently staining an independent set of samples with 5µM CFSE (Molecular Probes) in PBS for 5 min at room temperature. After three washes cells were lysed in 100µl 3% triton X-100 and fluorescence was determined as above.

All experiments were performed in triplicates, repeated at least three times and calculated as means±S.E. Values were expressed as relative light units (R.L.U.) or relative fluorescence units (R.F.U.), respectively, or as x-fold increases compared to controls. Statistical significances were calculated using the Student's *t* test (*, p<0.05).

### Expression and Functional Screening of a Retroviral cDNA Library

A retroviral cDNA expression library generated from RAW264.7 macrophages cloned into the retroviral vector pFB-Neo (Stratagene) was used for stable transduction of NIH3T3 cells as described [24]. Briefly, 1×10^6^ plat-E cells were transfected per 1µg DNA using FuGENE 6 transfection reagent (Roche Applied Science) according to the manufacturer's instruction and incubated for 24 hr at 37°C, followed by another 24 hr incubation at 32°C. Retroviral supernatants were harvested and used to transduce 1×10^5^ NIH3T3 cells in the presence of 5µg/ml polybrene (Sigma), which were then selected with 0.4mg/ml G418 (InvivoGen). The screening for cells expressing any mycobacterial-binding receptors was done by fluorescent microscopy as described in detail in [8] on a Zeiss inverted microscope Axiovert 40 CFL after incubation of the stably transfected NIH3T3 cells with *M.bovis* BCG expressing GFP prepared as described above. Positive cells were ring-isolated using cloning cylinders, expanded in culture for 4–7 days and re-screened until the majority of cells were found to be positive. Viral cDNA inserts were recovered from the genomic DNA of positive cells by conventional PCR using the primers 5′Retro and 3′pFB-Neo (Stratagene) and re-cloned into pFB-Neo for functional testing and sequencing.

### DNA Constructs and Generation of Stable Cell Lines

The 2.5kb cDNA of SRB1 was derived from the functional screen and subcloned into the retroviral vector pFB-Neo. Subcloning of SIGNR1 in pFB-Neo has been described before [25]. Stable NIH3T3 or R6F cells expressing SRB1 or SIGNR1 were generated by retroviral transduction as described above and grown in the presence of 0.4mg/ml G418 (InvivoGen).

### Western Blotting

Cells were lysed in Nonidet P-40 buffer (1% Nonidet P-40, 150mM NaCl, 10mM EDTA, 10mM NaN_3_, 10mM Tris-HCl (pH 8), 2mM Na_3_VO_4_, 10mM NaF, and complete EDTA-free protease inhibitors (Roche Applied Sciences) and incubated on ice for 30 min. Nuclei and cell debris were pelleted at 12000*g* for 20 min at 4°C, and supernatants were prepared for SDS-PAGE and Western blotting according to conventional protocols. Rabbit anti-mouse SR-B1 IgG (NB400-104, Novus Biologicals) and goat anti-rabbit IgG horseradish peroxidase conjugate (Jackson Immuno Research Laboratories, Inc.) were used to detect SR-B1 using ECL chemiluminescence substrate (Amersham Biosciences). As a loading control the blots were probed with a rabbit anti-mouse GAPDH monoclonal antibody (Cell Signaling Technology, Inc.).

### Flow Cytometry

Flow cytometry was performed according to standard protocols at 4°C. Briefly, adherent cells were lifted in 4mg/ml lidocaine hydrochloride (Sigma) in PBS supplemented with 10mM EDTA, washed in FACS wash solution (0.5% BSA in PBS) and placed at a density of 1×10^5^ cells in 96-well V-bottom plastic plates. Cells were blocked with 5% BSA in PBS or 5% heat-inactivated rabbit serum, respectively, and examined for the surface expression of SR-B1 or SIGNR1 using rabbit polyclonal anti-mouse SR-B1 IgG (NB400-113, Novus Biologicals) or rat monoclonal anti-mouse ERTR9 IgM (BMA Biomedicals) together with biotinylated mouse anti-rat IgM (Serotec), respectively. Rabbit IgG (Chromopure) or rat IgM (BD PharMingen), respectively, were used as isotype controls. Cells were stained with R-Phycoerythrin-conjugated donkey anti-rabbit IgG (Jackson Immuno Research Laboratories, Inc.) or R-Phycoerythrin-conjugated streptavidin (BD PharMingen), respectively, and fixed with 1% formaldehyde in PBS. The analysis was performed using a FACSCalibur (Becton Dickinson) together with the software CellQuest version 4. For the detection of ligand binding to SR-B1, 10µg/ml DiI-labelled LDL (Molecular Probes) was added to SR-B1 expressing R6F cells in serum-free DMEM for 2 hr at 37°C. Cells were then washed three times and lifted with 4mg/ml lidocaine hydrochloride (Sigma) in PBS supplemented with 10mM EDTA. Cells were spun down, resuspended in FACS wash solution containing 1% formaldehyde and analysed for ligand binding by FACS.

### Animals

C57BL/6 mice deficient in SR-B1 and its splice variant SR-B2 (*Scarb1^tm1Kri^*/*Scarb1^tm1Kri^*) were purchased from the Jackson Laboratory which were originally derived by Rigotti *et al.* [50]. Animals were maintained under specific pathogen-free conditions in the University of Cape Town animal facility. All work was approved by the Faculty of Health Sciences Animal Ethics Committee, University of Cape Town. As homozygous *Scarb1^tm1Kri^*/*Scarb1^tm1Kri^* female mice were not fertile, breeding had to be performed with heterozygous *Scarb1^tm1Kri^*/*Scarb1^+^* mice. However, the numbers of mutant offspring was significantly less than the expected Mendelian ratio of 1∶2∶1 as described earlier [50], [51], which significantly limited the number of animals available for experiments. Where indicated, mice were fed either a low cholesterol (LC, 0.15% cholesterol) or a high cholesterol (HC, 1.25% cholesterol) diet (Research Diets, Inc.) two weeks before and throughout the experiment. For these experiments, serum cholesterol levels were measured using the Cholesterol Enzymatic CHOD-PAP kit (KAT Laboratory & Medical (Pty) Ltd.).

### Isolation of Primary Macrophages

Resident or thioglycollate-elicited peritoneal macrophages as well as bone-marrow derived macrophages (BMDmØ) were isolated from 8–12 weeks old SR-B1^−/−^ mice or wild type C57BL/6 littermates by standard procedures. To isolate resident alveolar macrophages, lungs were lavaged with 1ml cold PBS (representing the bronchoalveolar lavage (BAL)), followed by repeated washes with 1ml PBS/10mM EDTA. Cells were maintained for 24 hr in RPMI (Invitrogen) with 10% heat-inactivated fetal calf serum (delta bioproducts), 2mM L-glutamine, 100U/ml penicillin and 100µg/ml streptomycin (Sigma) at 37°C, except for BMDmØ which were cultured in RPMI medium for 5–7 days supplemented with 20% (v/v) L-cell conditioned medium as a source of M-CSF. Binding experiments with these macrophages were performed in the absence of serum (and absence of M-CSF, in the case of BMDmØ) as described above. To determine the levels of TNF released into the supernatant, cells were washed after the binding assay in a parallel set of experiments, fresh DMEM containing 10% serum was added, and cells were incubated at 37°C. The amount of TNF was measured after 4 hr and 24 hr using the OptEIA murine TNFα ELISA kit (BD Biosciences) as described by the manufacturers, and normalized to cell number by CFSE staining. Data represent pooled values from at least 3 independent experiments.

### *M. tuberculosis* Infections

Frozen stocks of *Mycobacterium tuberculosis* H37Rv were thawed, briefly vortexed, and clumping disrupted by aspirating 30 times through a 29-gauge needle. The concentration was adjusted in sterile saline to deliver ∼100 CFU per mouse (low dose infection) or ∼1000 CFU per mouse (high dose infection). Appropriate numbers of 10–14 weeks old SR-B1^−/−^ male and female mice or wild type C57BL/6 littermates were then infected via the aerosol route using an inhalation exposure system (Glas-Col, Terre Haute, IN). In each experiment five mice were sacrificed 24 hr post-infection to confirm the infection dose. Animals were monitored throughout the experiments with regard to body weight and signs of disease. After 2 or 4 months, mice were sacrificed and lungs were removed and weighed. Defined tissue aliquots were homogenized in saline/0.04% Tween-80, and 10-fold serial dilutions were plated in duplicates onto Middlebrook 7H10 agar plates containing 10% OADC (Difco). Plates were incubated at 37°C, and colonies were counted after 3 weeks. Data are expressed as CFU per lung.

Lung homogenates were further analysed to measure the amounts of TNF, IFNγ, IL10, IL12p70 and IL6 by ELISA with appropriate OptEIA kits (BD Biosciences) as described by the manufacturers.

Lungs were also prepared for histology by fixing the big lobes in 4% phosphate-buffered formalin and then embedding in paraffin. 5 µm-thick sections were stained with haematoxylin and eosin (H&E) for evaluation of pathologic changes. The sizes of inflammatory lesions in four serial sections per Mtb infected mouse lung were determined by automated morphometry using a Nikon inverted microscope eclipse 90i and the software NIS-Elements BR 3.1 (Nikon).

## References

[1] Schafer G, Jacobs M, Wilkinson RJ, Brown GD (2009). Non-opsonic recognition of Mycobacterium tuberculosis by phagocytes.. J Innate Immun.

[2] Zimmerli S, Edwards S, Ernst JD (1996). Selective receptor blockade during phagocytosis does not alter the survival and growth of Mycobacterium tuberculosis in human macrophages.. Am J Respir Cell Mol Biol.

[3] Wieland CW, Koppel EA, den Dunnen J, Florquin S, McKenzie AN (2007). Mice lacking SIGNR1 have stronger T helper 1 responses to Mycobacterium tuberculosis.. Microbes Infect.

[4] Hu C, Mayadas-Norton T, Tanaka K, Chan J, Salgame P (2000). Mycobacterium tuberculosis infection in complement receptor 3-deficient mice.. J Immunol.

[5] Holscher C, Reiling N, Schaible UE, Holscher A, Bathmann C (2008). Containment of aerogenic Mycobacterium tuberculosis infection in mice does not require MyD88 adaptor function for TLR2, -4 and -9.. Eur J Immunol.

[6] Reiling N, Holscher C, Fehrenbach A, Kroger S, Kirschning CJ (2002). Cutting edge: Toll-like receptor (TLR)2- and TLR4-mediated pathogen recognition in resistance to airborne infection with Mycobacterium tuberculosis.. J Immunol.

[7] Reiling N, Klug K, Krallmann-Wenzel U, Laves R, Goyert S (2001). Complex encounters at the macrophage-mycobacterium interface: studies on the role of the mannose receptor and CD14 in experimental infection models with Mycobacterium avium.. Immunobiology.

[8] Schafer G, Brown GD (2009). Generation of retroviral macrophage cDNA expression libraries and functional screening for surface receptors.. Methods Mol Biol.

[9] Peiser L, Gordon S (2001). The function of scavenger receptors expressed by macrophages and their role in the regulation of inflammation.. Microbes Infect.

[10] Neyrolles O, Hernandez-Pando R, Pietri-Rouxel F, Fornes P, Tailleux L (2006). Is adipose tissue a place for Mycobacterium tuberculosis persistence?. PLoS ONE.

[11] Bowdish DM, Sakamoto K, Kim MJ, Kroos M, Mukhopadhyay S (2009). MARCO, TLR2, and CD14 are required for macrophage cytokine responses to mycobacterial trehalose dimycolate and Mycobacterium tuberculosis.. PLoS Pathog.

[12] Acton S, Rigotti A, Landschulz KT, Xu S, Hobbs HH (1996). Identification of scavenger receptor SR-BI as a high density lipoprotein receptor.. Science.

[13] Rhainds D, Brissette L (2004). The role of scavenger receptor class B type I (SR-BI) in lipid trafficking. defining the rules for lipid traders.. Int J Biochem Cell Biol.

[14] Connelly MA, Williams DL (2004). Scavenger receptor BI: a scavenger receptor with a mission to transport high density lipoprotein lipids.. Curr Opin Lipidol.

[15] Fidge NH (1999). High density lipoprotein receptors, binding proteins, and ligands.. J Lipid Res.

[16] Acton SL, Scherer PE, Lodish HF, Krieger M (1994). Expression cloning of SR-BI, a CD36-related class B scavenger receptor.. J Biol Chem.

[17] Rigotti A, Acton SL, Krieger M (1995). The class B scavenger receptors SR-BI and CD36 are receptors for anionic phospholipids.. J Biol Chem.

[18] Calvo D, Gomez-Coronado D, Lasuncion MA, Vega MA (1997). CLA-1 is an 85-kD plasma membrane glycoprotein that acts as a high-affinity receptor for both native (HDL, LDL, and VLDL) and modified (OxLDL and AcLDL) lipoproteins.. Arterioscler Thromb Vasc Biol.

[19] Philips JA, Rubin EJ, Perrimon N (2005). Drosophila RNAi screen reveals CD36 family member required for mycobacterial infection.. Science.

[20] Vishnyakova TG, Kurlander R, Bocharov AV, Baranova IN, Chen Z (2006). CLA-1 and its splicing variant CLA-2 mediate bacterial adhesion and cytosolic bacterial invasion in mammalian cells.. Proc Natl Acad Sci U S A.

[21] Voisset C, Callens N, Blanchard E, Op De Beeck A, Dubuisson J (2005). High density lipoproteins facilitate hepatitis C virus entry through the scavenger receptor class B type I.. J Biol Chem.

[22] Catanese MT, Graziani R, von Hahn T, Moreau M, Huby T (2007). High-avidity monoclonal antibodies against the human scavenger class B type I receptor efficiently block hepatitis C virus infection in the presence of high-density lipoprotein.. J Virol.

[23] Rodrigues CD, Hannus M, Prudencio M, Martin C, Goncalves LA (2008). Host scavenger receptor SR-BI plays a dual role in the establishment of malaria parasite liver infection.. Cell Host Microbe.

[24] Brown GD, Gordon S (2001). Immune recognition. A new receptor for beta-glucans.. Nature.

[25] Taylor PR, Brown GD, Herre J, Williams DL, Willment JA (2004). The role of SIGNR1 and the beta-glucan receptor (dectin-1) in the nonopsonic recognition of yeast by specific macrophages.. J Immunol.

[26] Gatfield J, Pieters J (2000). Essential role for cholesterol in entry of mycobacteria into macrophages.. Science.

[27] Stokes RW, Thorson LM, Speert DP (1998). Nonopsonic and opsonic association of Mycobacterium tuberculosis with resident alveolar macrophages is inefficient.. J Immunol.

[28] Martens GW, Arikan MC, Lee J, Ren F, Vallerskog T (2008). Hypercholesterolemia impairs immunity to tuberculosis.. Infect Immun.

[29] Kellner-Weibel G, de La Llera-Moya M, Connelly MA, Stoudt G, Christian AE (2000). Expression of scavenger receptor BI in COS-7 cells alters cholesterol content and distribution.. Biochemistry.

[30] Baranova I, Vishnyakova T, Bocharov A, Chen Z, Remaley AT (2002). Lipopolysaccharide down regulates both scavenger receptor B1 and ATP binding cassette transporter A1 in RAW cells.. Infect Immun.

[31] Randhawa AK, Ziltener HJ, Merzaban JS, Stokes RW (2005). CD43 is required for optimal growth inhibition of Mycobacterium tuberculosis in macrophages and in mice.. J Immunol.

[32] Van der Geize R, Yam K, Heuser T, Wilbrink MH, Hara H (2007). A gene cluster encoding cholesterol catabolism in a soil actinomycete provides insight into Mycobacterium tuberculosis survival in macrophages.. Proc Natl Acad Sci U S A.

[33] Pieters J (2008). Mycobacterium tuberculosis and the macrophage: maintaining a balance.. Cell Host Microbe.

[34] Schlesinger LS (1993). Macrophage phagocytosis of virulent but not attenuated strains of Mycobacterium tuberculosis is mediated by mannose receptors in addition to complement receptors.. J Immunol.

[35] Bulut Y, Michelsen KS, Hayrapetian L, Naiki Y, Spallek R (2005). Mycobacterium tuberculosis heat shock proteins use diverse Toll-like receptor pathways to activate pro-inflammatory signals.. J Biol Chem.

[36] Yadav M, Schorey JS (2006). The beta-glucan receptor dectin-1 functions together with TLR2 to mediate macrophage activation by mycobacteria.. Blood.

[37] Le Cabec V, Carreno S, Moisand A, Bordier C, Maridonneau-Parini I (2002). Complement receptor 3 (CD11b/CD18) mediates type I and type II phagocytosis during nonopsonic and opsonic phagocytosis, respectively.. J Immunol.

[38] Jo EK, Yang CS, Choi CH, Harding CV (2007). Intracellular signalling cascades regulating innate immune responses to Mycobacteria: branching out from Toll-like receptors.. Cell Microbiol.

[39] Feng CG, Scanga CA, Collazo-Custodio CM, Cheever AW, Hieny S (2003). Mice lacking myeloid differentiation factor 88 display profound defects in host resistance and immune responses to Mycobacterium avium infection not exhibited by Toll-like receptor 2 (TLR2)- and TLR4-deficient animals.. J Immunol.

[40] Drennan MB, Nicolle D, Quesniaux VJ, Jacobs M, Allie N (2004). Toll-like receptor 2-deficient mice succumb to Mycobacterium tuberculosis infection.. Am J Pathol.

[41] Abel B, Thieblemont N, Quesniaux VJ, Brown N, Mpagi J (2002). Toll-like receptor 4 expression is required to control chronic Mycobacterium tuberculosis infection in mice.. J Immunol.

[42] Stokes RW, Norris-Jones R, Brooks DE, Beveridge TJ, Doxsee D (2004). The glycan-rich outer layer of the cell wall of Mycobacterium tuberculosis acts as an antiphagocytic capsule limiting the association of the bacterium with macrophages.. Infect Immun.

[43] Hall-Stoodley L, Watts G, Crowther JE, Balagopal A, Torrelles JB (2006). Mycobacterium tuberculosis binding to human surfactant proteins A and D, fibronectin, and small airway epithelial cells under shear conditions.. Infect Immun.

[44] Ferguson JS, Voelker DR, McCormack FX, Schlesinger LS (1999). Surfactant protein D binds to Mycobacterium tuberculosis bacilli and lipoarabinomannan via carbohydrate-lectin interactions resulting in reduced phagocytosis of the bacteria by macrophages.. J Immunol.

[45] Ferguson JS, Voelker DR, Ufnar JA, Dawson AJ, Schlesinger LS (2002). Surfactant protein D inhibition of human macrophage uptake of Mycobacterium tuberculosis is independent of bacterial agglutination.. J Immunol.

[46] Sanchez MD, Garcia Y, Montes C, Paris SC, Rojas M (2006). Functional and phenotypic changes in monocytes from patients with tuberculosis are reversed with treatment.. Microbes Infect.

[47] Freeman AE, Price PJ, Igel HJ, Young JC, Maryak JM (1970). Morphological transformation of rat embryo cells induced by diethylnitrosamine and murine leukemia viruses.. J Natl Cancer Inst.

[48] Morita S, Kojima T, Kitamura T (2000). Plat-E: an efficient and stable system for transient packaging of retroviruses.. Gene Ther.

[49] Snewin VA, Gares MP, Gaora PO, Hasan Z, Brown IN (1999). Assessment of immunity to mycobacterial infection with luciferase reporter constructs.. Infect Immun.

[50] Rigotti A, Trigatti BL, Penman M, Rayburn H, Herz J (1997). A targeted mutation in the murine gene encoding the high density lipoprotein (HDL) receptor scavenger receptor class B type I reveals its key role in HDL metabolism.. Proc Natl Acad Sci U S A.

[51] Trigatti B, Rayburn H, Vinals M, Braun A, Miettinen H (1999). Influence of the high density lipoprotein receptor SR-BI on reproductive and cardiovascular pathophysiology.. Proc Natl Acad Sci U S A.

